# Stilbenoid gaylussacin modulates particulate matter-induced chromatin remodeling in macrophages to suppress chronic obstructive pulmonary disease

**DOI:** 10.1038/s41392-026-02579-7

**Published:** 2026-02-24

**Authors:** Jeong Yeon Sim, Jee Hwan Ahn, Hye-Young Min, Jae-Hwan Kwak, Suckchang Hong, Dae-Duk Kim, Ho-Young Lee

**Affiliations:** 1 Seoul National University, Seoul, Republic of Korea; 2 Chungbuk National University, Chungcheongbuk-do, Republic of Korea

**Keywords:** Respiratory tract diseases, Drug development

**Dear Editor**,

Chronic obstructive pulmonary disease (COPD), a leading cause of mortality worldwide, is characterized by chronic bronchitis and emphysema.^1^ Current treatment offers symptomatic relief but minimally affect disease progression.^2^ Cigarette smoke (CS) is the primary risk factor; but airborne particulate matter (PM) also contributes and synergizes with CS to accelerate disease progression. Dysregulated macrophages drive COPD pathogenesis by inducing inflammation and tissue destruction through excessive secretion of cytokines, reactive oxygen and nitrogen species (ROS/RNS), and proteases.^1^ We previously showed that PM exposure induces macrophage infiltration in the lungs of mice, wherein enhanced CCCTC-binding factor (CTCF) binding to the promoters of kynurenine pathway (KP) enzyme genes (*Kmo*, *Kynu*, *Haao*, and *Qprt*) reduces NAD^+^ synthesis, inactivates SIRT1, increases histone acetylation, and induces proinflammatory gene expression during COPD development.^3^ These effects were reversed by resveratrol, a stilbenoid SIRT1 activator.^3^

To explore the upstream mechanism of PM-induced CTCF activation, we profiled lung transcriptomes from PM-induced COPD mice. Hallmark gene sets enriched in PM-exposed lungs were linked to CK2, a known CTCF kinase (^**repository**^Fig. 1). In MH-S alveolar macrophages, PM exposure induced CK2 nuclear accumulation, CTCF threonine phosphorylation and its association with cohesin subunits (SMC1A, SMC3, and SA-1, but not SA-2), and chromatin binding of the CTCF-cohesin complex (^**repository**^Figs. 2–4). These effects were abolished by the CK2 inhibitor silmitasertib, which also restored the expression of KP enzyme and proinflammatory genes (^**repository**^Fig. 5) while suppressing PM-induced histone acetylation (^**repository**^Fig. 6). Lung tissues from mice with PM-induced COPD presented increased CTCF phosphorylation without changes in total CTCF expression (^**repository**^Fig. 7). Similar results were observed in CS-induced COPD,^4^ suggesting that aberrant CTCF activation is a common pathogenic mechanism driving COPD progression in both smokers and nonsmokers (^**repository**^Fig. 7). To evaluate clinical relevance, we analyzed publicly available single-cell RNA-sequencing data from COPD patients. Alveolar macrophages from COPD patients presented altered expression of CTCF target genes, including those in the KP (^**repository**^Fig. 8). These results indicate that CK2-dependent aberrant activation of CTCF drives macrophage-mediated inflammation in COPD.

Given the adverse effects, rapid metabolism, and photosensitivity of resveratrol, we sought safer and more effective inhibitors of COPD by screening stilbenoids (oxyresveratrol, pterostilbene, piceatannol, piceatannol 3’-O-glucoside, pinostilbene, rhapontin, [E]-2,3’,4,5’-tetramethoxystilbene, gaylussacin, and resveratrol) for their ability to inhibit PM-induced CTCF chromatin binding and oxidative stress in macrophages. Gaylussacin and pterostilbene showed the strongest suppression of CTCF chromatin binding (^**repository**^Fig. 9). Eight compounds reduced ROS production by >50% in PM-exposed MH-S cells; however, only gaylussacin inhibited both ROS and NO production without detectable cytotoxicity across multiple normal cell lines (MLE-12, RPE, MLg, and HT-22) (^**repository**^Fig. 10). Gaylussacin reduced PM-induced CTCF threonine phosphorylation and its association with cohesin subunits, as well as chromatin binding of the CTCF-cohesin complex (Fig. 1a; ^**repository**^Figs. 11–12), and restored NAD^+^ levels and SIRT1 activity (^**repository**^Fig. 13). In silico docking via Glide XP (Schrödinger Release 2024-2) predicted stronger binding of gaylussacin to CK2’s ATP-binding site (PDB: 1JWH) than to the CTCF-cohesin complex (PDB: 6QNX), with docking scores of –10.422 and –4.567, respectively (Fig. 1b; ^**repository**^Fig. 14). Structural modeling revealed that gaylussacin forms hydrogen bonds between its glycoside moiety and Leu45/Val116, a carboxyl interaction with Asp175, and a salt bridge with Lys68 of CK2 (Fig. 1b). In DARTS assays, gaylussacin protected CK2, but not CTCF, from proteolysis (^**repository**^Fig. 15). An in vitro kinase assay using recombinant CK2 protein demonstrated that gaylussacin directly inhibits CK2 kinase activity (^**repository**^Fig. 16). These results identify gaylussacin as a CK2 inhibitor that suppresses CTCF phosphorylation and CTCF–cohesin chromatin assembly, thereby restoring KP enzyme expression and SIRT1 activity in PM-exposed macrophages.

**Fig. 1:**
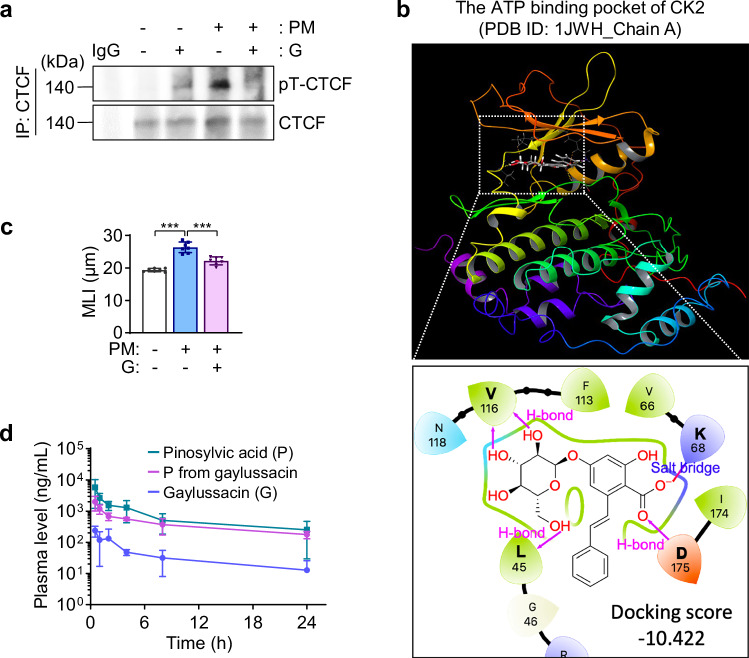
Discovery of gaylussacin as a potent, nontoxic CK2 inhibitor that suppresses PM-induced COPD development. **a** Regulation of CTCF phosphorylation by gaylussacin treatment. MH-S cells were exposed to particulate matter (PM; 50 μg/mL) for one month and then treated with PM in the presence or absence of gaylussacin (G; 10 μM) for 24 h. The final vehicle (DMSO) concentration was <0.1%. CTCF was immunoprecipitated from whole-cell lysates, followed by western blot analysis using a phospho-threonine antibody to assess CTCF threonine phosphorylation (pT-CTCF). **b** Docking model of gaylussacin within the ATP-binding pocket of CK2. The predicted binding mode illustrates potential hydrogen bonds and/or salt bridges between gaylussacin and the indicated amino acid residues within the ATP-binding pocket of CK2. **c** Therapeutic efficacy of gaylussacin in a murine model of PM-induced COPD. FVB mice were intratracheally instilled with PM (1.6 mg/kg) twice weekly for 4 weeks. Gaylussacin (G; 40 mg/kg, oral gavage, 5 days per week) was administered during weeks 3–4, following 2 weeks of PM exposure. Alveolar enlargement was quantified by mean linear intercept (MLI) in the indicated groups (mean ± SD, *n* = 7; ***p < 0.001, one-way ANOVA with Dunnett’s multiple-comparison test). **d** Pharmacokinetic profile of gaylussacin. Plasma concentration–time profiles of gaylussacin (G) and pinosylvic acid (P) following oral administration in FVB mice are shown (mean ± SD, *n* = 6). The plasma concentration of pinosylvic acid following gaylussacin administration is presented as “P from gaylussacin”

To identify the target cells of gaylussacin, flow cytometric analysis was performed on lung macrophages, fibroblasts, and epithelial cells from PM-exposed mice treated with vehicle or gaylussacin. Among these, macrophages presented the greatest PM-induced increase, which was significantly reduced by gaylussacin (^**repository**^Fig. 17). Furthermore, gaylussacin reversed PM-induced alterations in KP enzyme and proinflammatory gene expression in FACS-sorted macrophages, but not in epithelial cells or fibroblasts (^**repository**^Fig. 18). These results indicate that macrophages represent the predominant cellular target of gaylussacin. We then evaluated the therapeutic efficacy of gaylussacin in a murine model of PM-induced COPD. Four weeks of PM exposure induced COPD-like pathology characterized by alveolar enlargement, matrix metalloproteinase (MMP) activation, apoptosis, oxidative stress, mucus hypersecretion, and elevated proinflammatory cytokine levels in the bronchoalveolar lavage fluid (BALF), all of which were suppressed by oral gaylussacin treatment initiated at week 2 (Fig. 1c; ^**repository**^Figs. 19–20). Treatment during the 2-week withdrawal period alone or from week 2 through withdrawal also prevented PM-induced lung deterioration (^**repository**^Fig. 21). Kinase assays revealed that CK2 activity was significantly increased in PM-exposed lungs and effectively suppressed by gaylussacin treatment (^**repository**^Fig. 19). These findings indicate that gaylussacin mitigates PM-induced inflammation and COPD progression primarily through macrophage-targeted inhibition of the CK2/CTCF pathway.

Pharmacokinetic analysis of gaylussacin-treated mice revealed plasma detection of its aglycone, pinosylvic acid, indicating in vivo deglycosylation (Fig. 1d). Both gaylussacin and equimolar pinosylvic acid reached peak plasma levels within 1 h; however, pinosylvic acid presented a longer half-life and slower clearance, suggesting greater metabolic stability and bioavailability. In PM-induced COPD mice, both compounds reduced COPD progression (^**repository**^Fig. 22) without adverse effects on body weight or serum ALT, AST, and BUN levels (^**repository**^Fig. 23). Thus, gaylussacin and pinosylvic acid are well tolerated and effectively alleviate PM-induced COPD through anti-inflammatory actions.

In summary, our results show that CK2-mediated CTCF phosphorylation and chromatin remodeling drive macrophage-driven inflammation during PM-induced COPD progression. The aglycone pinosylvic acid exhibits superior pharmacokinetic properties and efficacy but lower aqueous solubility than gaylussacin. Overall, gaylussacin is a promising therapeutic candidate for mitigating macrophage-driven inflammation in PM-induced COPD, although its in vivo efficacy in PM-exposed mice may not be solely macrophage specific. Future studies investigating the role of CTCF in PM-induced COPD employing patient tissues with well-documented PM exposure, as well as clinical evaluation of the efficacy, pharmacokinetics, and safety of gaylussacin in COPD patients or high-risk populations, are warranted.

## Supplementary information

Supplementary Information

Supplementary Materials

## Data Availability

The data supporting the findings of this study are provided in the article and in the supplementary file deposited in the Zenodo online repository (10.5281/zenodo.17866881), where the datasets are labeled as ^repository^Fig. [figure number] in the main text. Additional raw data are available from the corresponding author upon reasonable request.
