# Supplementary material for: Stilbenoid gaylussacin modulates particulate matter-induced chromatin remodeling in macrophages to suppress chronic obstructive pulmonary disease
Source: Signal Transduct Target Ther. 2026 Feb 24;11:62. doi: 10.1038/s41392-026-02579-7 (PMC12929684; doi:10.1038/s41392-026-02579-7)
Supplement: Supplementary file 1 — Supplementary Information [file 41392_2026_2579_MOESM1_ESM.docx]

Supplementary Materials for

Stilbenoid gaylussacin modulates particulate matter-induced chromatin remodeling in macrophages to suppress chronic obstructive pulmonary disease

Jeong Yeon Sim, Jee Hwan Ahn, Hye-Young Min, Jae-Hwan Kwak, Suckchang Hong, Dae-Duk Kim, Ho-Young Lee

Correspondence to: [hylee135@snu.ac.kr](mailto:hylee135@snu.ac.kr)

**This PDF file includes:**

Materials and Methods

Materials and Methods

* Additional methods are available in the online repository supplementary file that has been deposited in Zenodo (https://doi.org/10.5281/zenodo.17866881).

Reagents

Particulate matter (PM, cat no. Standard Reference Material [SRM] 1648a) was acquired from the National Institute of Standards and Technology (NIST, Gaithersburg, MD, USA). Gaylussacin and pinosylvic acid were synthesized according to a previous report^1^. Other stilbenoids were purchased from MedChemExpress (Monmouth Junction, NJ, USA). Fluorescein-conjugated DQ-gelatin, fluorochrome-conjugated secondary antibodies, acetonitrile (ACN; HPLC grade), ethanol (EtOH; purity: 99.5%), and water (HPLC grade) were purchased from Thermo Fisher Scientific (Waltham, MA, USA). Dimethyl sulfoxide (DMSO) was purchased from Sigma-Aldrich (St. Louis, MO, USA) or Daejung Chemicals & Metals Co., Ltd. (Siheung, Republic of Korea). Losartan (LST) potassium (internal standard (IS); purity ≥ 99.5%), polyethylene glycol 400 (PEG400), and other reagents, unless specified otherwise, were procured from Sigma-Aldrich. PM was suspended in phosphate-buffered saline (PBS) at a concentration of 10 mg/mL by sonication. Stock and working solutions of other compounds used in in vitro experiments were prepared in DMSO. For in vivo experiments, gaylussacin and pinosylvic acid were dissolved in 50% PEG400 in normal saline (NS, acquired from Daihan Pharm. Co., Ltd. [Seoul, Republic of Korea]) or distilled water.

Animal experiments

All in vivo experiments were performed in accordance with the protocols approved by the Institutional Animal Care and Use Committee of Seoul National University. Animal care and experimental procedures adhered to the ethical guidelines of Seoul National University. Mice were housed in a controlled environment under a 12-hour light/dark cycle at constant temperature and humidity, with free access to standard chow and water. Six- to twelve-week-old male and female FVB/N (FVB) mice (Japan SLC, Inc., Hamamatsu, Shizuoka, Japan) were randomly assigned to each experimental group and intratracheally exposed to either PBS or PM (SRM 1648a, NIST; 1.6 mg/kg) twice a week for two or four weeks. Gaylussacin (40 mg/kg) and pinosylvic acid (24.48 mg/kg) were administered orally once daily for four weeks. After completion of treatment, mice were euthanized by isoflurane overdose followed by exsanguination via cardiac puncture. The lungs were perfused with ice-cold PBS, excised, and either snap-frozen, embedded in OCT compound (Sakura Finetek USA Inc., Torrance, CA, USA), or fixed in 4% paraformaldehyde (PFA) for subsequent analyses. When required, bronchoalveolar lavage fluid (BALF) was collected following the final treatment by flushing the lungs twice with 1.5 mL of ice-cold PBS after perfusion with ice-cold PBS. The in vivo toxicity of the drug using blood chemistry was assessed using a veterinary hematology analyzer (Fuji DRI-Chem 3500s; Fujifilm, Tokyo, Japan) in accordance with the manufacturer's guidelines.

Histological analyses, in situ zymography, terminal deoxynucleotidyl transferase dUTP nick end labeling (TUNEL) staining, and dihydroethidium (DHE) staining

Whole-lung images were obtained using an automated multimodal tissue imaging system (Vectra; PerkinElmer, Alameda, CA, USA). The degree of alveolar enlargement was assessed by calculating the mean linear intercept (MLI) using ImageJ software (version 1.54g; National Institutes of Health [NIH], Bethesda, MD, USA)^2-4^. Periodic acid–Schiff (PAS) staining was performed according to the previously established protocol^5^. For in situ zymography, frozen lung tissue blocks were sectioned, air-dried, and incubated with fluorescein-conjugated dye-quenched (DQ) gelatin diluted in low–melting point agarose for 3 h at 37 ℃. TUNEL staining of lung cryosections was performed using a commercial assay kit (Millipore, Billerica, MA, USA) according to the manufacturer’s instructions. For DHE staining, unfixed cryosections were incubated with 5 μM DHE (Thermo Fisher Scientific) for 30 min at 37 °C and counterstained with DAPI. Fluorescence images were captured using a Zeiss LSM700 laser scanning confocal microscope (Carl Zeiss SMT GmbH, Oberkochen, Germany).

Chromatin fractionation

Chromatin fractions were prepared by using a Subcellular Protein Fractionation kit (Thermo Fisher Scientific) according to the manufacturer’s instructions.

Cell culture

The murine alveolar macrophage cell line (MH-S) was maintained in RPMI 1640 medium (Welgene, Gyeongsan, Republic of Korea) supplemented with 10% fetal bovine serum (FBS; Welgene), 1% antibiotics–antimycotics (Welgene), and 50 µM β-mercaptoethanol (Thermo Fisher Scientific). The source and culture conditions of various normal cells are shown in our previous publication^6^. Cells were cultured at 37 °C in a humidified atmosphere containing 5% CO_2_. PM-treated MH-S cells were established by continuous exposure to standard reference PM (SRM 1648a, NIST) at a final concentration of 50 µg/mL for 1 month. Cells maintained for less than 3 months after thawing or receipt without mycoplasma contamination were used.

CK2 activity assay

The effect of gaylussacin on CK2 activity using recombinant CK2 protein (LSBio) and lung tissue lysates was determined using a CycLex CK2 kinase assay/inhibitor screening kit (Medical & Biological Laboratories, Tokyo, Japan) according to the manufacturer’s protocol.

Molecular docking

The molecular docking study was performed using Glide in Extra Precision (XP), employed by Schrödinger (Release 2024-2) in accordance with previous studies^7^. The protein structures of the CTCF-SA2-SCC1 complex and ATP-binding pocket of CK2 were obtained from the Protein Data Bank (PDB) with IDs 6QNX and 1JWH_Chain A, respectively. The two-dimensional structure of gaylussacin was acquired in SDF file format using CS ChemDraw (version 20) and transformed into a three-dimensional structure. The docking score was computed to evaluate the binding affinity of gaylussacin for the aforementioned proteins.

Pharmacokinetics of gaylussacin and pinosylvic acid in FVB mice

The pharmacokinetic profiles of gaylussacin and pinosylvic acid after oral administration in FVB mice were evaluated using the retro-orbital blood collection method at predetermined time points (0 as a blank, 30, 60, 120, 240, 480, and 1440 min). The processing of collected blood samples, chromatographic analysis using an HPLC–MS/MS system, and pharmacokinetic parameter estimation were performed as previously described^6^.

Statistical analysis

Data are expressed as mean ± standard deviation (SD). Each in vitro experiment was independently repeated at least twice, and representative results are presented. The values shown in the graphs represent replicates from a representative experiment. Statistical methods are described in the corresponding figure legends. Statistical significance was defined as *p* < 0.05.

**References**

1 Song, I. et al. First Total Synthesis of Gaylussacin and Its Stilbene Derivatives. *J Nat Prod* **84**, 1366-1372 (2021).

2 Mitzner, W. Use of mean airspace chord length to assess emphysema. *J Appl Physiol (1985)* **105**, 1980-1981 (2008).

3 Noh, M. et al. Particulate matter-induced metabolic recoding of epigenetics in macrophages drives pathogenesis of chronic obstructive pulmonary disease. *J Hazard Mater* **464**, 132932 (2024).

4 Bouchery, T. et al. The Study of Host Immune Responses Elicited by the Model Murine Hookworms Nippostrongylus brasiliensis and Heligmosomoides polygyrus. *Curr Protoc Mouse Biol* **7**, 236-286 (2017).

5 Gu, W. et al. B7-H3 participates in the development of Asthma by augmentation of the inflammatory response independent of TLR2 pathway. *Sci Rep* **7**, 40398 (2017).

6 Min, H. Y. et al. Gaylussacin, a stilbene glycoside, inhibits chronic obstructive pulmonary disease in mice. *Redox Biol* **85**, 103744 (2025).

7 Jung, Y. J. et al. Microwave-Promoted Total Synthesis of Puniceloid D for Modulating the Liver X Receptor. *Molecules* **29** (2024).
