# Supplementary material for: Stilbenoid gaylussacin modulates particulate matter-induced chromatin remodeling in macrophages to suppress chronic obstructive pulmonary disease
Source: Signal Transduct Target Ther. 2026 Feb 24;11:62. doi: 10.1038/s41392-026-02579-7 (PMC12929684; doi:10.1038/s41392-026-02579-7)
Supplement: Supplementary file 2 — Supplementary Materials [file 41392_2026_2579_MOESM2_ESM.pdf]

Additional online supplementary materials for

Stilbenoid gaylussacin modulates particulate matter-induced chromatin remodeling in macrophages to suppress chronic obstructive pulmonary disease

Jeong Yeon Sim, Jee Hwan Ahn, Hye-Young Min, Jae-Hwan Kwak, Suckchang Hong, Dae-Duk Kim, Ho-Young Lee

This pdf file includes:

- Materials and Methods
- Online repository supplementary figures ([repository]Fig. 1–23)

Materials and Methods

Immunoprecipitation and co-immunoprecipitation (IP/co-IP)

Cells were lysed in ice-cold EBC buffer (50 mM Tris-HCl [pH 8.0], 120 mM NaCl, 0.5% NP-40, 1 mM EDTA, and protease/phosphatase inhibitors) for 30 min on ice and then subjected to centrifugation at 13,000 rpm for 15 min at 4 °C. Equal amounts of total protein (approximately 1 mg) were incubated overnight at 4 °C with 1 µg of the indicated antibodies (anti-CTCF [Cell Signaling Technology, Danvers, MA, USA) or anti-SA-1 [Santa Cruz Biotechnology, Dallas, TX, USA]) under gentle rotation, followed by incubation with protein A-agarose beads (Merck KGaA, Darmstadt, Germany) for 2 h at 4 °C to capture immune complexes. Beads were washed three times with ice-cold EBC buffer and eluted by boiling in 5× SDS sample buffer for 5 min at 99 °C. The immunoprecipitated proteins were separated by SDS–PAGE and analyzed by western blot analysis.

Western blot analysis and Immunofluorescence staining

Western blotting was carried out using chromatin fractions and lysates derived from lung tissues or cultured cells. Lysates were prepared with a modified RIPA lysis buffer (20 mM Tris-HCl [pH 7.4] 150 mM NaCl, 1 mM EDTA, 1 mM EGTA, 1% Triton X-100, 1 mM Na_3VO_4, 10 mM sodium pyrophosphate [NaPP], 20 mM β-glycerophosphate, 1 mM phenylmethylsulfonyl fluoride [PMSF], 1 µg/mL aprotinin, and 1 µg/mL leupeptin), in accordance with the procedures outlined in our recent publication[1]. We employed 8–15% sodium dodecyl sulfate-polyacrylamide gel electrophoresis (SDS-PAGE) for protein separation, followed by the electrotransfer of proteins onto polyvinylidene fluoride (PVDF) membranes. Membranes were incubated in a blocking buffer consisting of 3% bovine serum albumin (BSA) in Tris-buffered saline (TBS) with 0.1% Tween-20 (TBST) for 1 h at room temperature. After incubating membranes with primary antibodies diluted in blocking buffer at a ratio of 1:1,000 overnight at 4°C, they were washed multiple times with TBST. Subsequently, membranes were incubated with secondary antibodies (CellNest, Hanam-si, Gyeonggi-do, Republic of Korea) diluted in 3% nonfat dry milk in TBST at a ratio of 1:5,000–1:10,000 for 1 h at room temperature. Membranes were washed multiple times with TBST, and blots were detected using an enhanced chemiluminescence kit (WestGlow Femto ECL chemiluminescent substrate; Biomax, Guri-si, Gyeonggi-do, Republic of Korea). The following primary antibodies were utilized: anti-CTCF (Cell Signaling), anti-SA-1 (Santa Cruz), anti-SA-2 (STAG2; Proteintech, Rosemont, IL, USA), anti-SMC1A (Proteintech), anti-SMC3 (Proteintech), anti-phospho-Thr (Santa Cruz), anti-CK2α (Santa Cruz), anti-β-actin (Santa Cruz), anti-Lamin B1 (Abcam, Cambridge, UK), pan-acetyl (Santa Cruz), H3K27Ac (ABclonal, Woburn, MA, USA), H4K8Ac (Active Motif, Carlsbad, CA, USA), Histone H3 (Cell Signaling), Histone H4 (Cell Signaling), β-tubulin (Cell Signaling), and GAPDH (Cell Signaling). Densitometric analysis was performed using ImageJ software (National Institutes of Health, Bethesda, MD, USA).

The detailed procedure for IF staining was presented in our previous study[1]. Fixed cryosections underwent treatment with 4% paraformaldehyde, followed by permeabilization with 0.2% Triton X-100 in TBS for 10 min at ambient temperature. Subsequently, sections were blocked with 5% normal serum in TBS for 1 h at ambient temperature, followed by incubation with anti-CK2 primary antibody (Santa Cruz; 1:100) diluted in 1% BSA in TBS overnight at 4 °C. After washing with wash solution (TBS containing 0.025% Triton X-100), slides were incubated

with fluorochrome-conjugated secondary antibodies for 1 h at ambient temperature. After several washes with wash buffer, the slides were counterstained with 4′,6-diamino-2-phenylindole (DAPI) and mounted using a mounting solution (Thermo Fisher Scientific, Waltham, MA, USA).

<u>Real-time PCR</u>

Total RNA was extracted using RNAiso Plus reagent (Takara Bio Inc., Shiga, Japan) according to the manufacturer's instructions. cDNA was synthesized from 1 µg of total RNA using the First-Strand cDNA Synthesis Kit (TransGen Biotech, Beijing, China). Real-time PCR was performed using the TOPreal™ SYBR Green qPCR PreMIX kit (Enzynomics, Daejeon, Republic of Korea) on a QuantStudio™ 5 Real-Time PCR System (Thermo Fisher Scientific). Relative gene expression levels were calculated using the comparative cycle threshold (ΔΔCt) method[2]. The following primers were used for real-time PCR (5′–3′):

Kmo: F- GCACTGAATGCCTGCTTTCTTGC; R- CCAGACCAATGGCTTTCAAGGC
Kynu: F- GTATGCGGATGGTAAAGCCACG; R- CACTGAACAGGATCACGGCGAT
Haao: F- GGTTCCACTGTAAGGACCTTGG; R- AGGGTTGGGTTTTCCTGTTCGG
Qprt: F- GAAAGACAACCATGTAGTGGCGG; R- GGCTGCTACATTCCACCTCTAC
Il6: F- CTCTGGGAAATCGTGGAAAT; R- CCAGTTTGGTAGCATCCATC
Tnf: F- GGTGCCTATGTCTCAGCCTCTT; R- GCCATAGAACTGATGAGAGGGAG
Cxcl1: F- TCCAGAGCTTGAAGGTGTTGCC; R- AACCAAGGGAGCTTCAGGGTCA
Ccl2: F- TTAAAAACCTGGATCGGAACCAA; R- GCATTAGCTTCAGATTTACGGGT
Actb (set 1): F- TGTCCACCTTCCAGCAGATGT; R- AGCTCAGTAACAGTCCGCCTAG
Actb (set 2): F- GGCTGTATTCCCCTCCATCG; R- CCAGTTGGTAACAATGCCATGT

<u>Cell viability assay</u>

Cells (2 × 10^3 cells per well in 96-well plates) were treated with vehicle (DMSO) or test compounds (10 µM) for two days. Cell viability was assessed using the crystal violet assay. Briefly, cells were fixed with 100% methanol and air-dried, followed by staining with 0.025% crystal violet for 30 min at room temperature. After several washes with tap water, the stained cells were solubilized in 1% SDS solution, and absorbance was measured at 570 nm. Cell viability was calculated by normalizing the absorbance values of each compound-treated group to those of the vehicle-treated control group. The final concentration of vehicle (DMSO) was <0.1%.

<u>Measurement of cellular ROS levels and nitric oxide production</u>

MH-S cells exposed to PM (50 µg/mL) for 1 month were treated with either vehicle (DMSO) or test compounds (10 µM), in the presence or absence of PM (50 µg/mL), for 24 h. For measurement of intracellular ROS levels, cells were incubated with 20 µM H_2DCF-DA at 37 °C for 30 min, and fluorescence intensity was recorded using a plate fluorometer (SpectraMax M5, Molecular Devices, San Jose, CA, USA) with excitation at 485 nm and emission at 530 nm. Nitric oxide production was quantified using a nitrite assay based on the Griess reaction, following the instructions (Promega Technical Bulletin #TB229).

Measurement of NAD^+ production and SIRT1 activity

NAD^+ and NADH production and SIRT1 activity were quantified using commercially available assay kits (all from Abcam), following the protocols provided by the manufacturer.

DARTS assay

The DARTS assay was conducted according to previously published methods[3]. Purified recombinant CK2 protein (acquired from LSBio, Newark, CA, USA) was treated with gaylussacin (10, 50, and 100 μM) for 1 h at RT and then further incubated with proteinase K (PK, a 1:500 ratio) for 10 min at 4 °C. The PK reaction was stopped by adding 5× SDS sample buffer and incubating for 5 min at 99 °C, followed by SDS-PAGE and western blot analysis.

ELISA

ELISA for the levels of IL-17A, TNF-α, and IL-6 in the bronchoalveolar lavage fluid (BALF) was performed using a commercially available kit (all from Thermo Fisher Scientific) according to the manufacturer's instructions.

Flow cytometry analysis and cell sorting

Detailed procedures for single cell preparation from murine lung tissues and FACS are described in our previous publication[4]. We utilized the lung tissue from FVB mice that were treated intratracheally with PM, either alone or together with gaylussacin (40 mg/kg) for two weeks. For FACS of lung cell populations, the following antibodies were used: anti-CD45 (APC/Cy7; BioLegend, San Diego, CA, USA; 1:100), F4/80 (PE/Cy7; BioLegend; 1:100), CD31 (PE/Cy7; BioLegend; 1:100), EpCAM (PE; Thermo Fisher Scientific; 1:100), and PDGFRα (APC; Thermo Fisher Scientific; 1:100). Macrophages were identified as $CD45^+F4/80^+$ cells, epithelial cells as $EpCAM^+$ within the $CD45^-CD31^-$ fraction, and fibroblasts as $PDGFR\alpha^+$ within the $CD45^-CD31^-$ fraction. Cell sorting was performed on a BD FACSAria II system (BD Biosciences, San Jose, CA, USA).

For further discrimination of the level of immune cell subpopulations, epithelial cells, and fibroblasts, lung single-cell suspensions were stained for surface markers including CD45 (APC; BioLegend; 1:100), CD4 (APC; BioLegend; 1:400), CD8a (PE; BioLegend; 1:200), F4/80 (PE/Cy7; BioLegend, 1:50), CD11b (APC; BioLegend, 1:500), Ly6G (PE; BioLegend, 1:100), PDGFRα (APC; Thermo Fisher Scientific; 1:100), and EpCAM (PE; Thermo Fisher Scientific; 1:100) for 30 min at 4 °C. Flow cytometric acquisition was performed using a BD FACSLyric flow cytometers (BD Biosciences). Data were analyzed using FlowJo software (Tree Star, Ashland, OR, USA). All staining and sorting procedures were conducted on ice or at 4 °C.

Gene set enrichment analysis (GSEA)

In accordance with previously published literature[5] and the GSEA user guide accessible on the GSEA homepage (https://docs.gsea-msigdb.org/#GSEA/GSEA_User_Guide/GSEA), we conducted GSEA using GSEA software (version 4.3.3, UC San Diego and Broad Institute), employing hallmark gene sets sourced from the molecular signature database (MSigDB), along with the RNA sequencing data of PM-treated murine lungs documented in our previous publication[1] and archived in a public repository (Gene Expression Omnibus [GEO], accession number GSE235860).

In silico analysis

Publicly available single-cell RNA sequencing (scRNA-seq) data were obtained from the GEO database under accession number GSE136831. Cell cluster annotations were adopted from the original publications associated with the dataset. For downstream analysis, macrophage populations were selected based on these published cluster labels. Data processing and analysis were performed using the Seurat package (version 5.2.1) in R. Raw gene expression counts were log-normalized using the NormalizeData function, and cells were filtered and clustered following the standard Seurat workflow. Gene expression patterns were visualized using dot plots generated with ggplot2 (version 4.0.0) in R. CTCF-regulated genes included in the analysis were selected based on previously published studies[6,7].

References

1 Noh, M. et al. Particulate matter-induced metabolic recoding of epigenetics in macrophages drives pathogenesis of chronic obstructive pulmonary disease. *J Hazard Mater* **464**, 132932 (2024).

2 Livak, K. J. & Schmittgen, T. D. Analysis of relative gene expression data using real-time quantitative PCR and the 2(-Delta Delta C(T)) Method. *Methods* **25**, 402-408 (2001).

3 Lomenick, B. et al. Target identification using drug affinity responsive target stability (DARTS). *Proc Natl Acad Sci U S A* **106**, 21984-21989 (2009).

4 Min, H. Y. et al. Gaylussacin, a stilbene glycoside, inhibits chronic obstructive pulmonary disease in mice. *Redox Biol* **85**, 103744 (2025).

5 Subramanian, A. et al. Gene set enrichment analysis: a knowledge-based approach for interpreting genome-wide expression profiles. *Proc Natl Acad Sci U S A* **102**, 15545-15550 (2005).

6 Nikolic, T. et al. The DNA-binding factor Ctcf critically controls gene expression in macrophages. *Cell Mol Immunol* **11**, 58-70 (2014).

7 Yang, X. et al. CTCF is selectively required for maintaining chromatin accessibility and gene expression in human erythropoiesis. *Genome Biol* **26**, 44 (2025).

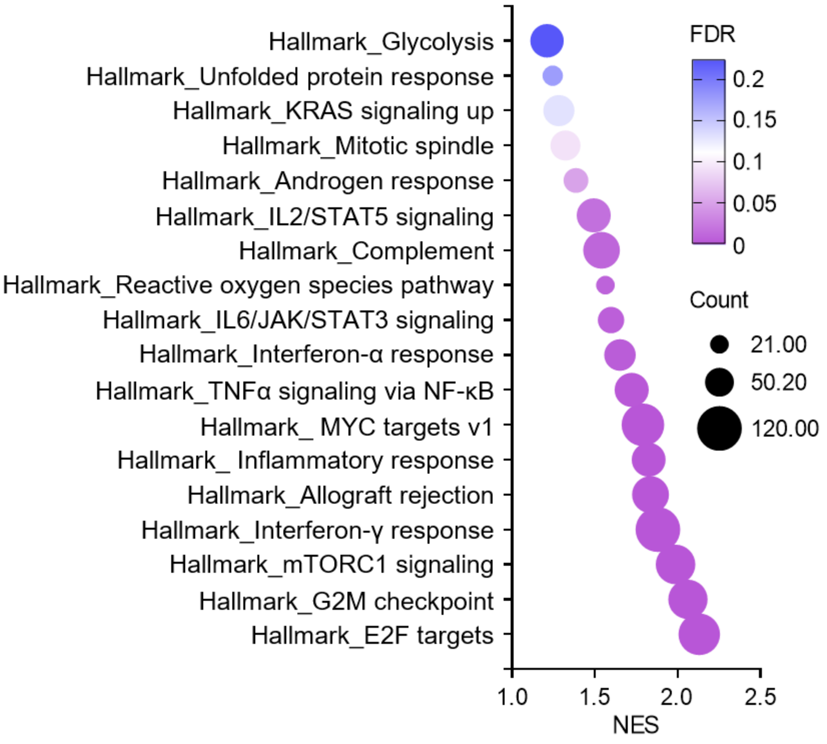

repository**Fig. 1. Enrichment of Hallmark gene sets in the lungs of mice exposed to PM.** Gene set enrichment analysis (GSEA) was performed to examine Hallmark gene set enrichment in the lungs of mice exposed to particulate matter (PM) for four weeks compared with vehicle (PBS)-treated controls. RNA-sequencing data are archived in the Gene Expression Omnibus (GEO) under accession number GSE235860.

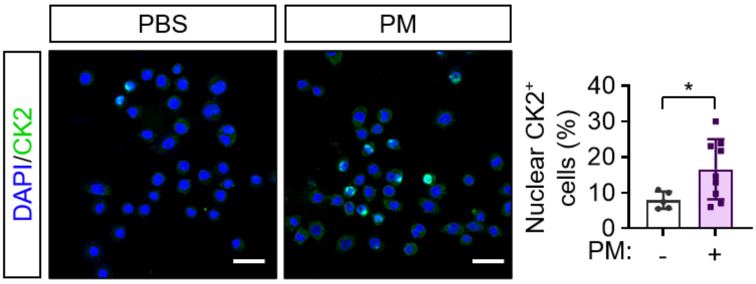

repository**Fig. 2. Increased nuclear translocation of CK2 following PM exposure in mouse alveolar macrophage cells.** MH-S cells were exposed to PM (50 μg/mL) for one month and subsequently treated with PM in the presence or absence of silmitasertib (Sil, 10 μM) for 24 h. Representative immunofluorescence images and quantitative analysis of CK2α nuclear translocation are shown (mean ± SD, n = 5 or 9; *p < 0.05, as determined by two-tailed Welch's t-test). The final vehicle (DMSO) concentration was <0.1%. Scale bars: 20 μm.

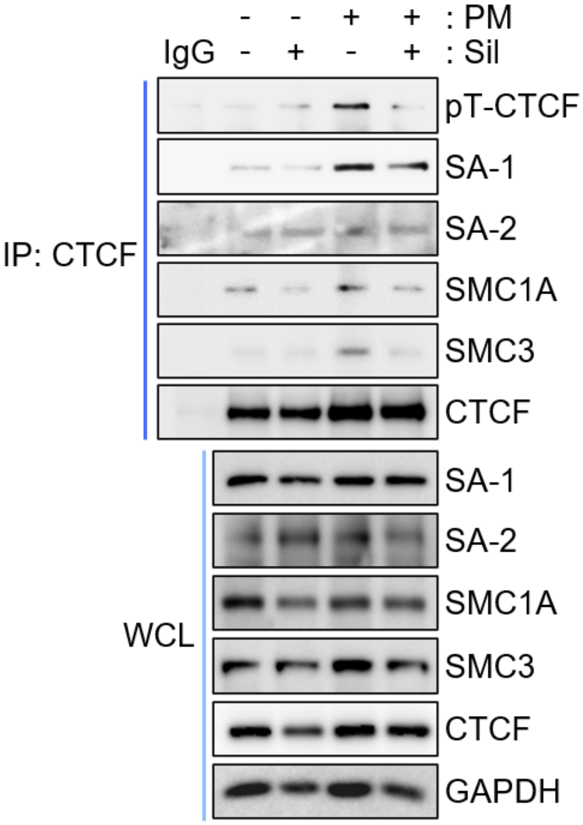

repository**Fig. 3. Inhibition of PM-induced CTCF phosphorylation and disruption of the PM-induced interaction between CTCF and cohesin subunits by CK2 blockade in mouse alveolar macrophages.** MH-S cells were exposed to particulate matter (PM; 50 μg/mL) for one month and subsequently treated with PM in the presence or absence of silmitasertib (Sil; 10 μM) for 24 h. The final vehicle (DMSO) concentration was <0.1%. Immunoprecipitation (IP) of CTCF from whole-cell lysates (WCLs), followed by western blot analysis of the indicated proteins, was performed to assess CTCF threonine phosphorylation and its interaction with cohesin subunits.

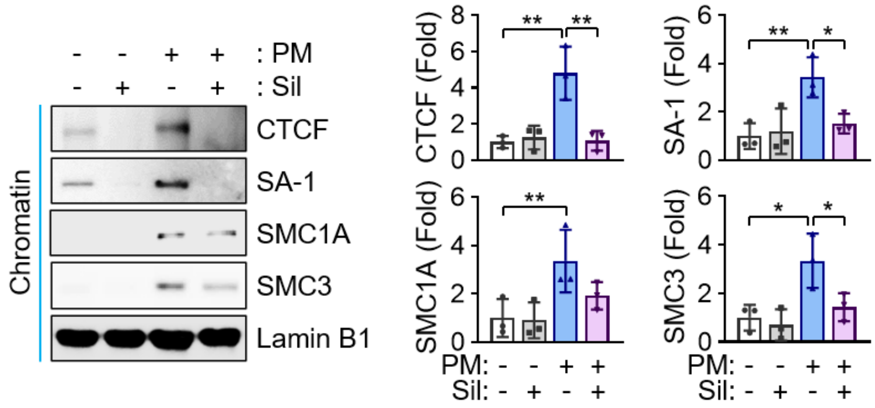

repository**Fig. 4. Inhibition of PM-induced increases in chromatin binding of CTCF and cohesin subunits by CK2 blockade.** Representative western blot images and quantification of western blot analyses of chromatin-bound CTCF and cohesin subunits in MH-S cells following PM (50 μg/mL) exposure with or without silmitasertib (Sil, 10 μM; mean ± SD, n = 3; *p < 0.05; **p < 0.01, as determined by one-way ANOVA with Dunnett's multiple comparison test). The final vehicle (DMSO) concentration was <0.1%.

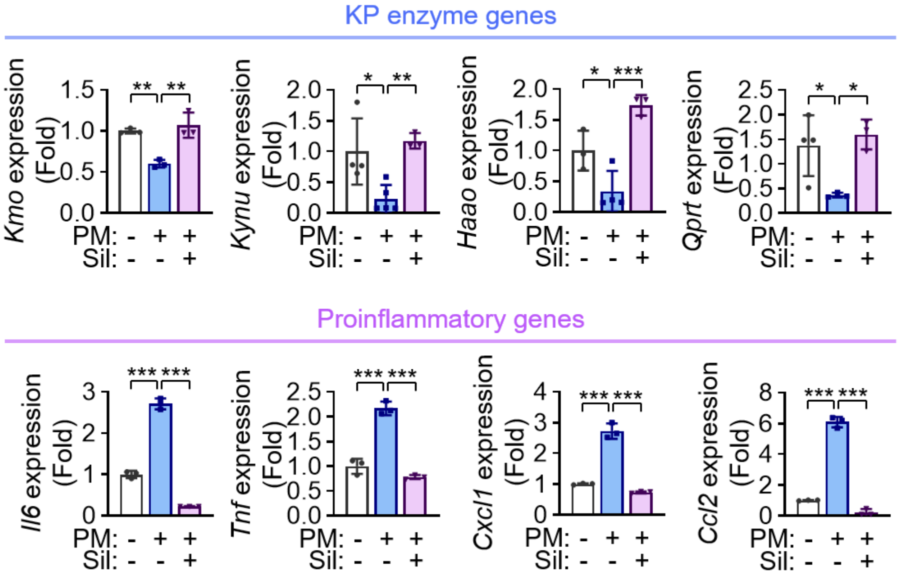

[repository]**Fig. 5. Restoration of PM-mediated modulation of KP enzyme gene expression and proinflammatory gene expression in mouse alveolar macrophages.** MH-S cells were exposed to PM (50 μg/mL) for one month and then treated with PM in the presence or absence of silmitasertib (Sil, 10 μM) for 24 h. The final vehicle (DMSO) concentration was <0.1%. Real-time PCR analysis of kynurenine pathway (KP) enzyme genes (*Kmo*, *Kynu*, *Haao*, and *Qprt*) and proinflammatory genes (*Il6*, *Tnf*, *Cxcl1*, and *Ccl2*) is shown (mean ± SD, n = 3–4; *$p < 0.05$; **$p < 0.01$; ***$p < 0.001$, as determined by one-way ANOVA with Dunnett's multiple comparison test).

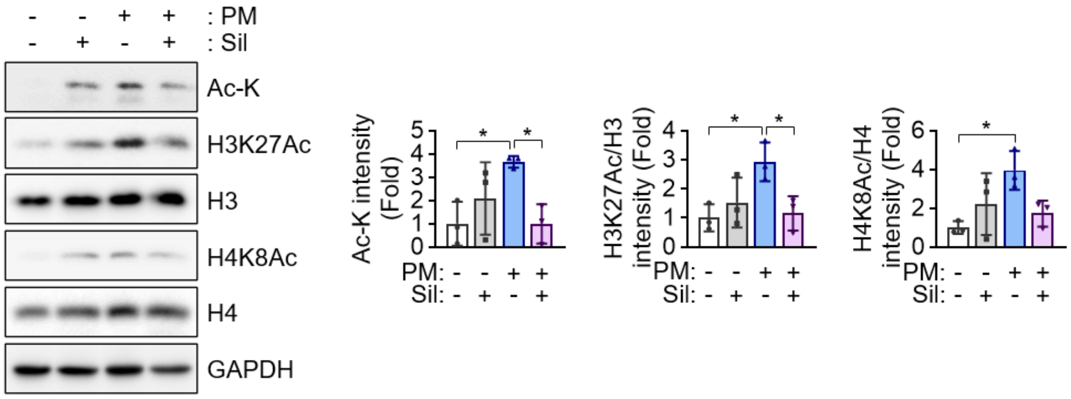

repository**Fig. 6. Inhibition of PM-induced histone acetylation by CK2 blockade.** Western blot analysis of the specified proteins in MH-S cells treated with PM (50 μg/mL) in the absence or presence of silmitasertib (Sil, 10 μM) for 24 h and quantitative analyses of the indicated protein expression levels (mean ± SD, $n = 3$; $^{*}p < 0.05$, as determined by one-way ANOVA with Dunnett's multiple comparison test). The final vehicle (DMSO) concentration was <0.1%.

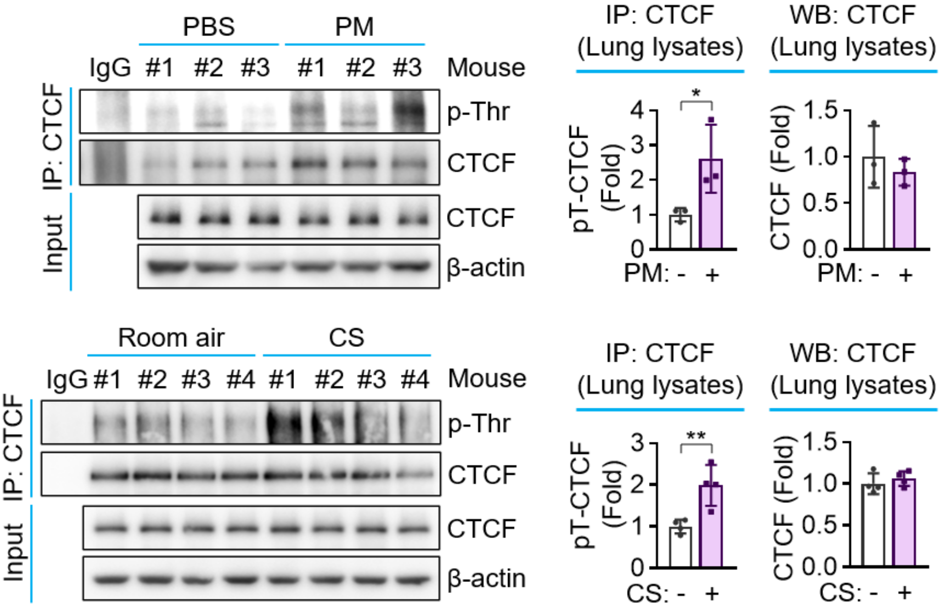

repositoryFig. 7. **Upregulation of CTCF phosphorylation in lung tissues of mice exposed to particulate matter or cigarette smoking.** Representative western blot images and quantification of western blot analyses of CTCF immunoprecipitates for phosphorylated CTCF at threonine residues (pT-CTCF) and corresponding input proteins in lung tissue lysates from mice exposed to particulate matter (PM; mean ± SD, n = 3) or cigarette smoke (CS; mean ± SD, n = 4) for four weeks (*$p < 0.05$; **$p < 0.01$, as determined by a two-tailed Student's t test).

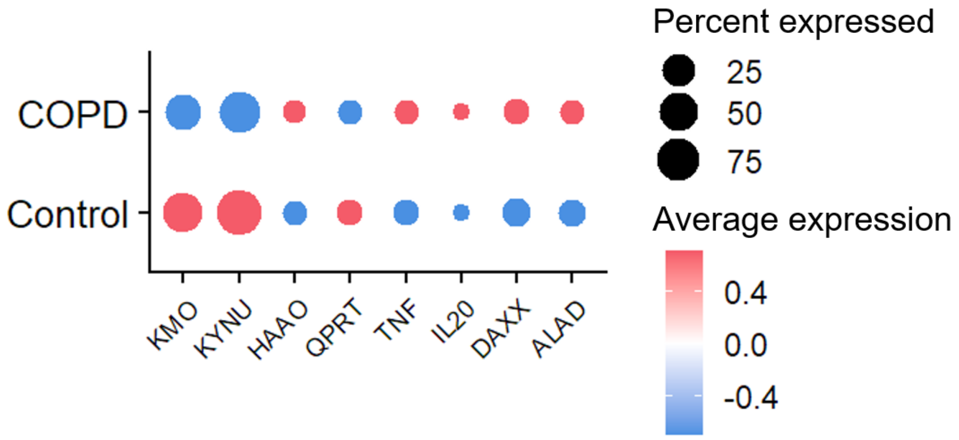

repository**Fig. 8. Modulation of kynurenine pathway gene expression and CTCF target gene expression in the alveolar macrophage cluster of lungs from patients with COPD compared with healthy controls.** Analysis of the publicly available single-cell RNA-sequencing dataset (GSE136831) demonstrating modulation of KP enzyme genes (*KMO*, *KYNU*, *HAAO*, and *QPRT*) and additional CTCF target genes (*TNF*, *IL20*, *DAXX*, and *ALAD*) in alveolar macrophage clusters.

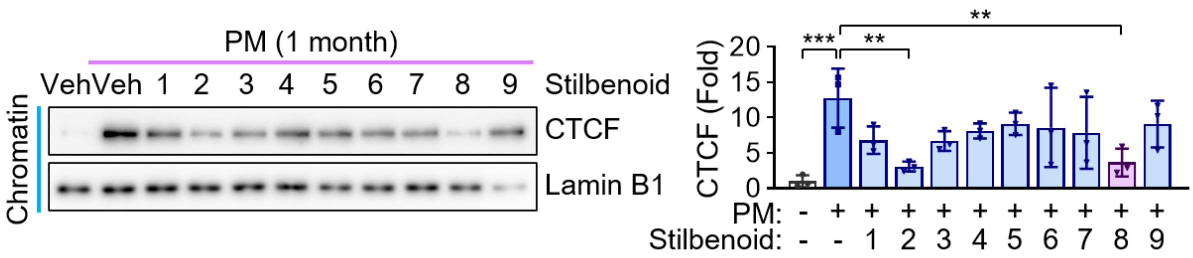

repository**Fig. 9. Modulation of PM-induced CTCF binding on chromatin by stilbenoid treatment in MH-S cells.** Representative western blot images and quantitative analysis illustrating changes in chromatin-bound CTCF after PM exposure with or without stilbenoid treatment. MH-S cells were exposed to PM (50 μg/mL) for one month and then treated with PM for 24 h in the absence or presence of the indicated stilbenoids (10 μM): **1**: oxyresveratrol; **2**: pterostilbene; **3**: piceatannol; **4**: piceatannol-3′-O-glucoside; **5**: pinostilbene; **6**: rhapontin; **7**: [E]-2,3′,4,5′-tetramethoxystilbene (TMS); **8**: gaylussacin; **9**: resveratrol (mean ± SD, n = 3; ** $p < 0.01$; *** $p < 0.001$, as determined by one-way ANOVA with Dunnett's multiple comparison test). The final vehicle (DMSO) concentration was <0.1%.

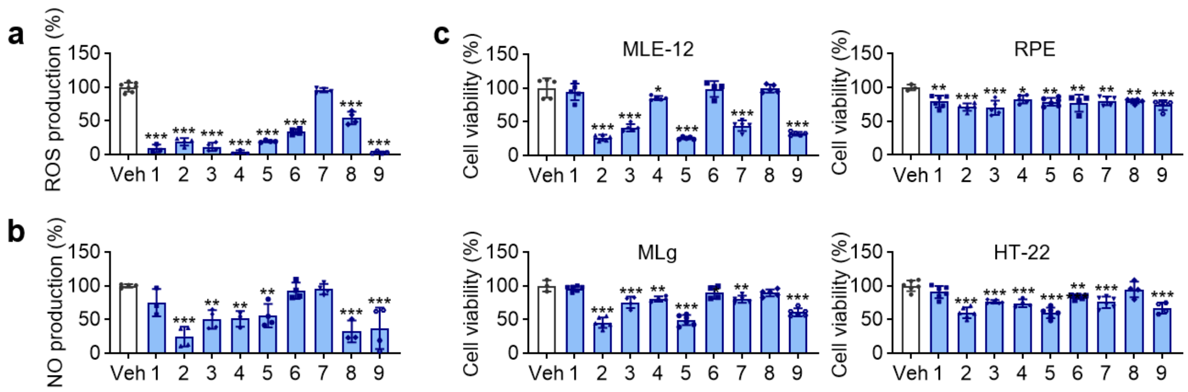

[repository]Fig. 10. Modulation of cellular ROS and nitric oxide (NO) production in MH-S cells and viability of normal cells following stilbenoid treatment. (**a, b**) MH-S cells exposed to PM (50 µg/mL) for one month were treated with the indicated stilbenoids for 24 h. Stilbenoids tested: **1**: oxyresveratrol; **2**: pterostilbene; **3**: piceatannol; **4**: piceatannol 3'-O-glucoside; **5**: pinostilbene; **6**: rhapontin; **7**: [E]-2,3',4,5'-tetramethoxystilbene (TMS); **8**: gaylussacin; **9**: resveratrol. (**a**) Cellular ROS levels (mean ± SD, n = 3–7). (**b**) NO production (mean ± SD, n = 3–4). (**c**) Crystal violet assay showing effects on the viability of normal cells after 2-day treatment (mean ± SD, n = 3–6). *$p < 0.05$; **$p < 0.01$; ***$p < 0.001$, as determined by one-way ANOVA with Dunnett's multiple comparison test. The final vehicle (DMSO) concentration was <0.1%.

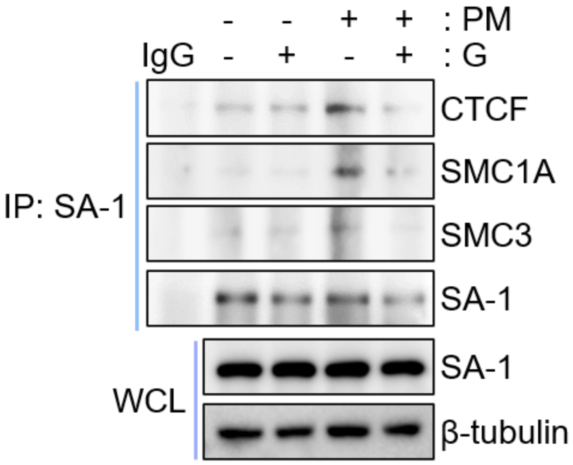

repository**Fig. 11. Inhibition of PM-induced interaction between CTCF and cohesin subunits by gaylussacin treatment in mouse alveolar macrophages.** MH-S cells were exposed to particulate matter (PM; 50 μg/mL) for one month and subsequently treated with PM in the presence or absence of gaylussacin (G; 10 μM) for 24 h. The final vehicle (DMSO) concentration was <0.1%. Immunoprecipitation (IP) of SA-1 from whole-cell lysates (WCLs), followed by western blot analysis of the indicated proteins, was performed to assess the interaction between CTCF and cohesin subunits.

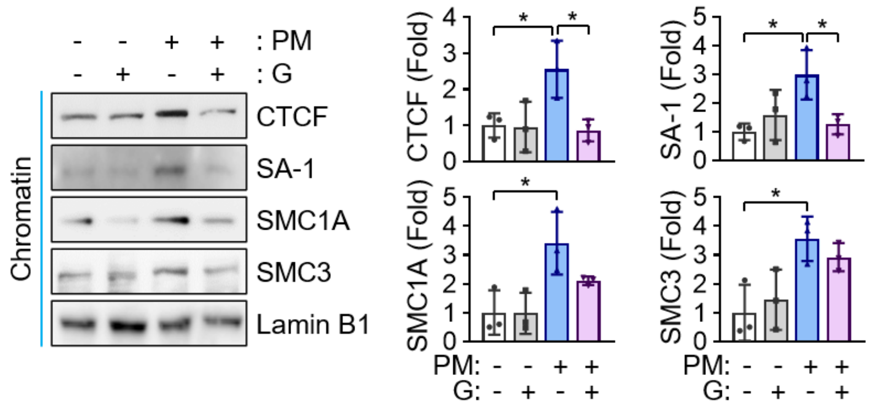

repository**Fig. 12. Inhibition of PM-induced increases in chromatin binding of CTCF and cohesin subunits by gaylussacin treatment.** Representative western blot images and quantification of western blot analyses of chromatin-bound CTCF and cohesin subunits in MH-S cells following PM (50 μg/mL) exposure in the presence or absence of gaylussacin (G; 10 μM; mean ± SD, n = 3; *p < 0.05, as determined by one-way ANOVA with Dunnett's multiple comparison test). The final vehicle (DMSO) concentration was <0.1%.

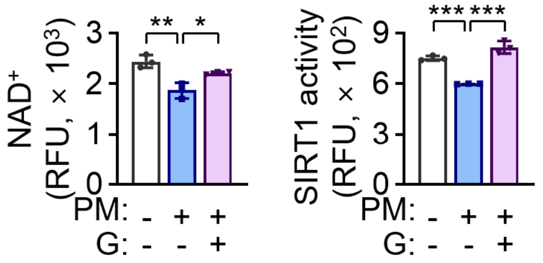

repository**Fig. 13. Restoration of PM-induced decreases in NAD$^+$ production and SIRT1 activity by gaylussacin treatment.** MH-S cells exposed to 50 μg/mL PM for one month and then treated with PM in the absence or presence of gaylussacin (G, 10 μM) for 24 h. NAD$^+$ production and SIRT1 activity were measured by using commercially available kits (mean ± SD, n = 3; *p < 0.05; **p < 0.01; ***p < 0.001, as determined by one-way ANOVA with Dunnett's multiple comparison test).

The CTCF/SA2/SCC1 complex (PDB ID: 6QNX)

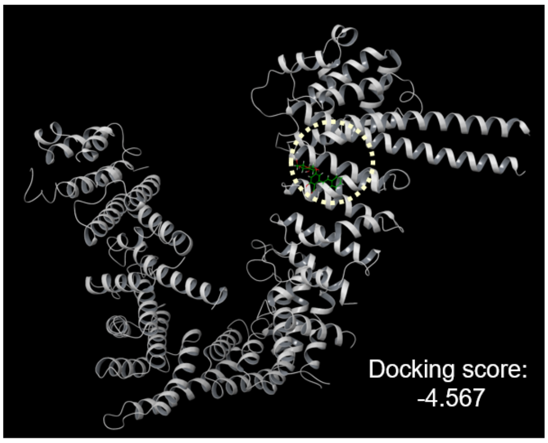

[repository]**Fig. 14. In silico docking analysis predicting the binding mode of gaylussacin to the CTCF/SA2/SCC1 complex.** Shown is the predicted docking pose and the docking score for the binding mode.

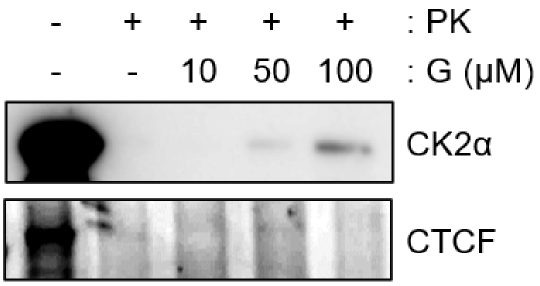

[repository]**Fig. 15. Gaylussacin binding to CK2 protein.** Drug affinity responsive target stability (DARTS) experiments demonstrate protection of CK2, but not CTCF, against proteinase K (PK)-mediated proteolysis after treatment with gaylussacin (G).

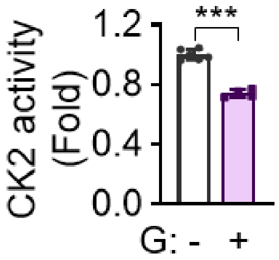

repository**Fig. 16. Inhibition of CK2 enzymatic activity by gaylussacin treatment.** In vitro kinase assay using recombinant CK2 protein to evaluate direct inhibition of CK2 enzymatic activity by gaylussacin (G) treatment (mean ± SD, n = 4 or 6; ***p < 0.001, as determined by a two-tailed Student's t test).

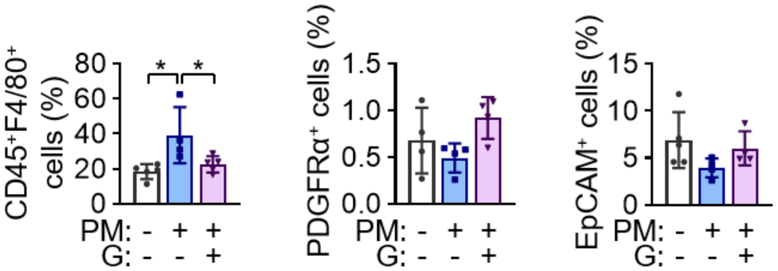

repository**Fig. 17. Modulation of F4/80⁺ macrophages, PDGFRα⁺ fibroblasts, and EpCAM⁺ epithelial cells in the lungs of mice following PM exposure, with or without gaylussacin treatment.** FVB mice received intratracheal instillation of PM (1.6 mg/kg) twice weekly for two weeks, with or without gaylussacin (G; 40 mg/kg, oral gavage, five days/week). Flow cytometric analysis of $CD45^{+}F4/80^{+}$ macrophages, PDGFRα^{+} fibroblasts, and $EpCAM^{+}$ epithelial cells in the lungs of PM-exposed mice with or without gaylussacin (G) treatment is shown (mean ± SD, n = 4–5; $^{*}p < 0.05$, as determined by one-way ANOVA with Dunnett's multiple comparison test).

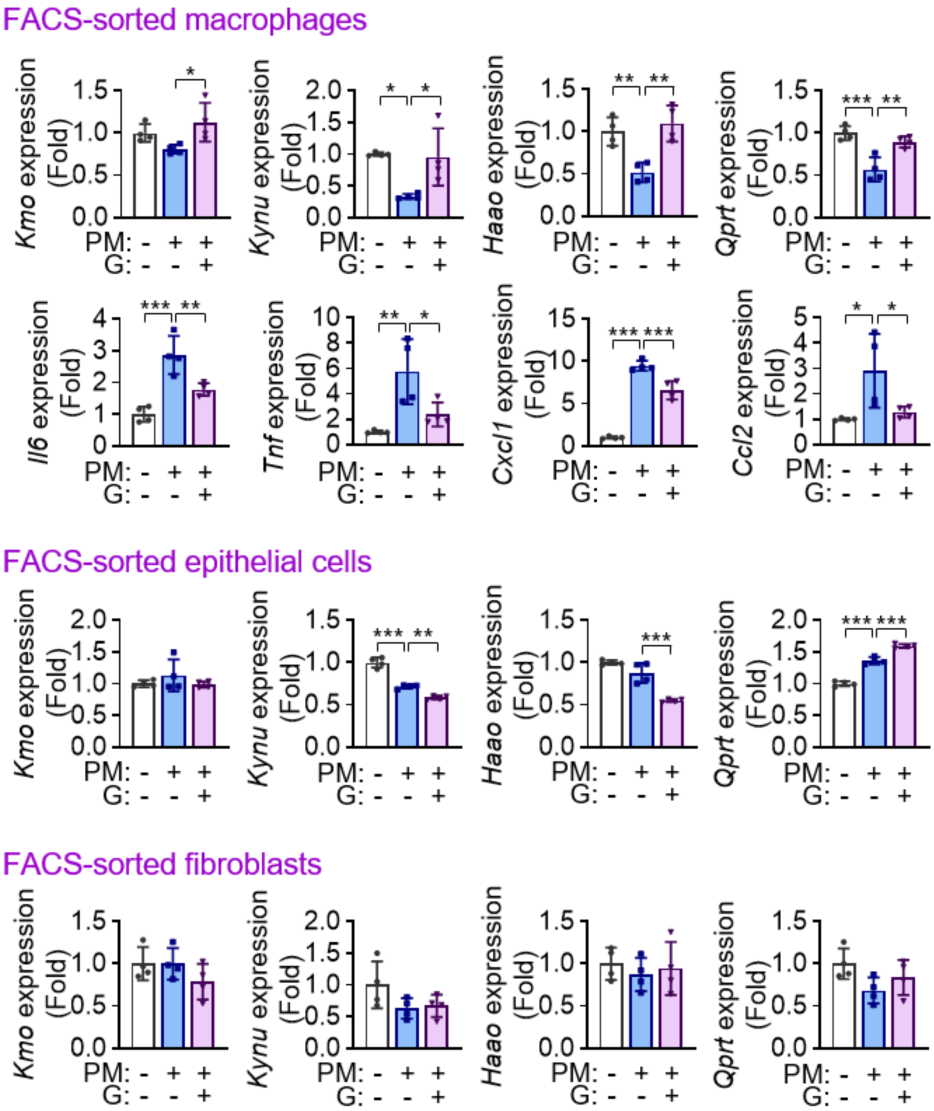

repository**Fig. 18. Modulation of KP enzyme gene expression and proinflammatory gene expression in the lungs of PM-exposed mice following gaylussacin treatment.** Real-time PCR analysis of KP enzyme genes (*Kmo*, *Kynu*, *Haao*, and *Qprt*) and proinflammatory genes (*Il6*, *Tnf*, *Cxcl1*, and *Ccl2*) was performed using FACS-sorted macrophages, epithelial cells, and fibroblasts isolated from murine lungs exposed to PM (1.6 mg/kg), either alone or in combination with gaylussacin (G; 40 mg/kg) for two weeks (mean ± SD, n = 3–4; *p < 0.05; **p < 0.01; ***p < 0.001, as determined by one-way ANOVA with Dunnett's multiple comparison test).

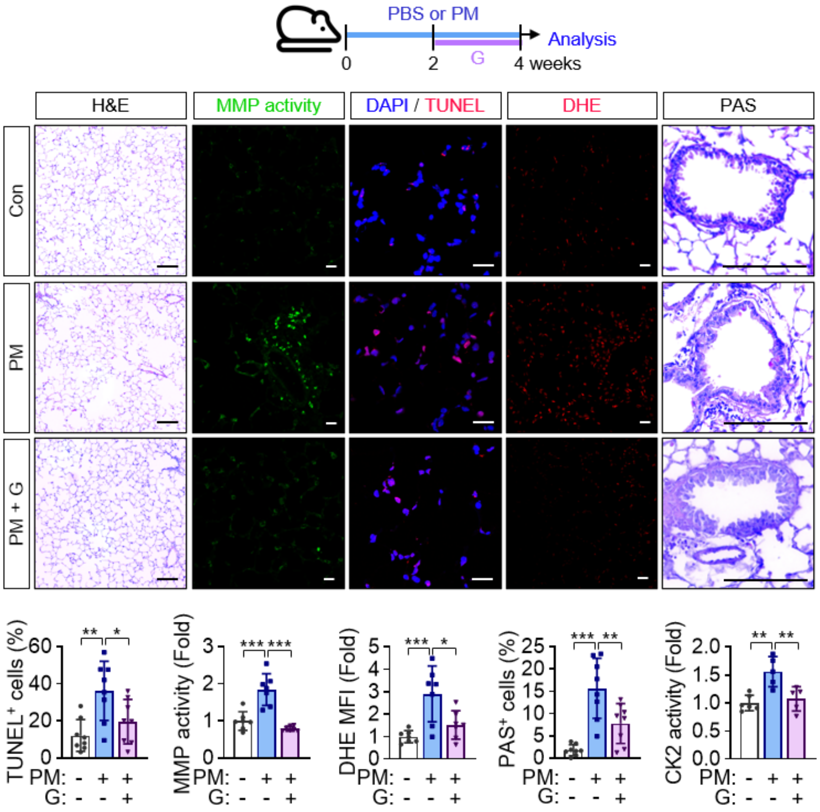

repositoryFig. 19. **Alleviation of PM-induced COPD pathogenesis via oral administration of gaylussacin in mice.** FVB mice were exposed to PM (1.6 mg/kg) twice a week for four weeks by intratracheal instillation. Gaylussacin (G, 40 mg/kg) was administered orally five days a week for two weeks following exposure to PM for two weeks. Shown are the experimental design, representative images, and quantification of matrix metalloproteinase (MMP) activity, apoptosis (TUNEL), reactive oxygen species (ROS) production (dihydroethidium [DHE]), and mucus secretion (Periodic acid–Schiff [PAS]) in the indicated groups (mean ± SD, n = 7–8; *p < 0.05; **p < 0.01; ***p < 0.001, one-way ANOVA with Dunnett's multiple comparison test). CK2 enzymatic activity in murine lung tissues was assessed by a CK2 kinase assay using lung lysates (mean ± SD, n = 5–6; **p < 0.01, as determined by one-way ANOVA with Dunnett's multiple comparison test). The mouse icon used for drawing the schematic diagram was retrieved from Flaticon (www.flaticon.com). Scale bars: 100 μm (H&E and PAS staining); 20 μm (in situ zymography, TUNEL, and DHE staining).

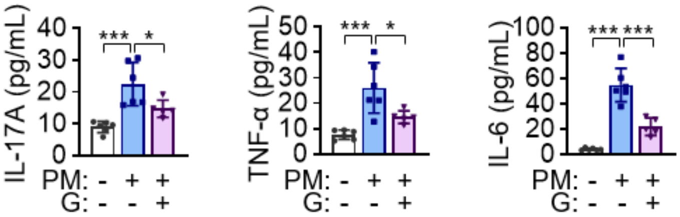

repository**Fig. 20. Modulation of proinflammatory cytokines in PM-exposed mice treated with or without gaylussacin treatment.** ELISA quantification of IL-17A, TNF-α, and IL-6 in the bronchoalveolar lavage fluid (BALF) from mice exposed to PM (1.6 mg/kg), either alone or together with gaylussacin (G; 40 mg/kg) (mean ± SD, n = 5–6; *$p < 0.05$; ***$p < 0.001$, as determined by one-way ANOVA with Dunnett's multiple comparison test).

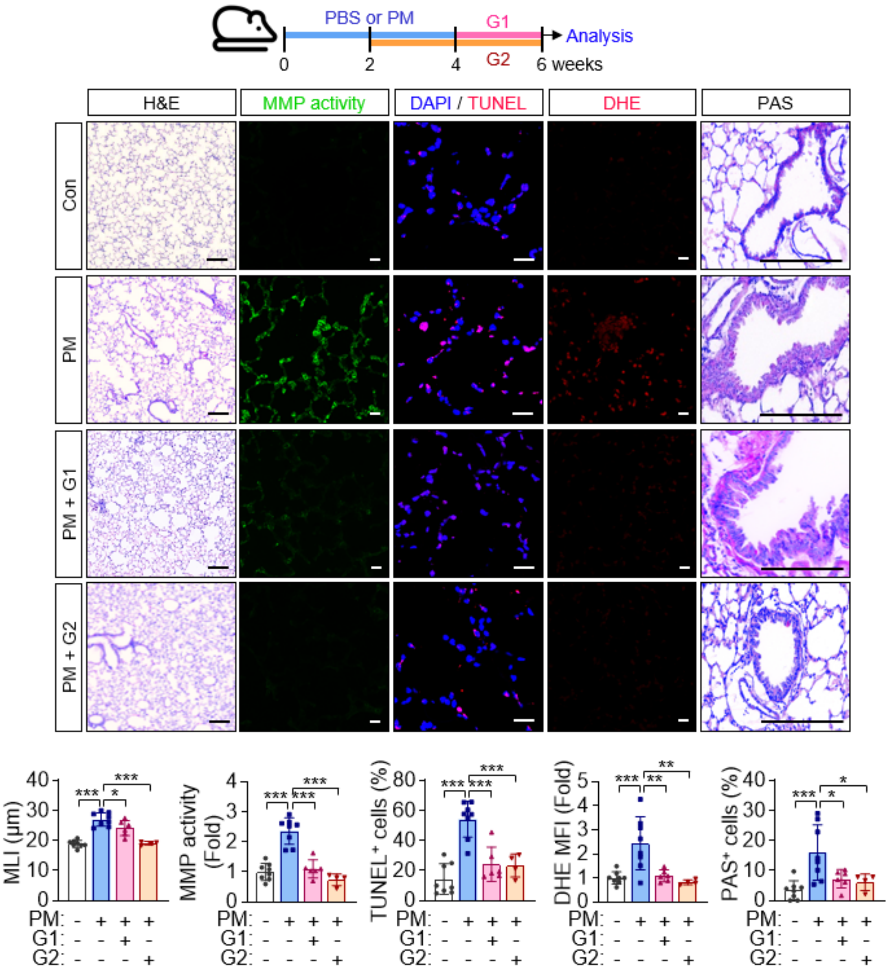

repositoryFig. 21. Alleviation of PM-induced COPD-like pathology by oral administration of gaylussacin in mice. FVB mice were exposed to PM (1.6 mg/kg) for four weeks by intratracheal instillation. Gaylussacin (40 mg/kg) was administered orally for two weeks following PM exposure (G1) or for two weeks during PM exposure plus an additional two weeks post-exposure (G2). Shown are the experimental design, representative images, and quantitative analyses of MLI, MMP activity (in situ zymography, normalized to mean fluorescence intensity of the vehicle group), $TUNEL^+$ cells (normalized to $DAPI^+$ cells), DHE staining (normalized to vehicle group MFI), and PAS staining (normalized to $hematoxylin^+$ bronchiolar cells) (mean ± SD, n = 4–8; *$p < 0.05$; **$p < 0.01$; ***$p < 0.001$, as determined by one-way ANOVA with Dunnett's multiple comparison test). The mouse icon used for drawing the schematic diagram was retrieved from Flaticon (www.flaticon.com). Scale bars: 100 μm (H&E and PAS staining); 20 μm (in situ zymography, TUNEL, and DHE staining).

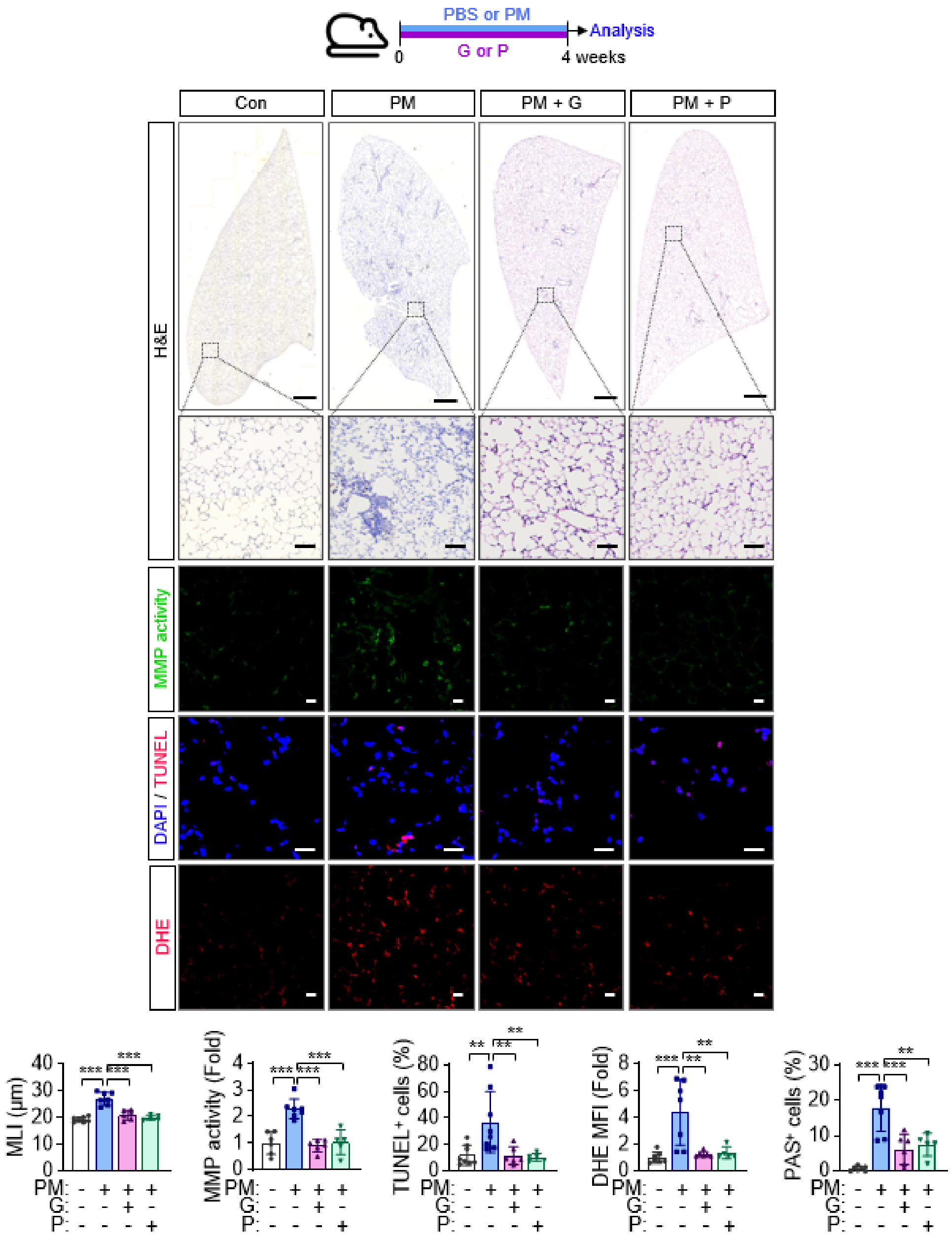

repository**Fig. 22. In vivo efficacy of gaylussacin versus pinosylvic acid.** FVB mice were exposed to PM twice a week for four weeks by intratracheal instillation, either alone or together with gaylussacin or pinosylvic acid (96 μmol/kg; 40 mg/kg gaylussacin [G] or 24.48 mg/kg pinosylvic acid [P], respectively) by oral administration (once a day, five days/week). Shown are the experimental design, representative images, and quantification of alveolar enlargement (MLI), MMP activity, apoptosis (TUNEL), ROS production (DHE), and mucus secretion (PAS) in the indicated groups (mean ± SD, n = 5–8; *$p < 0.05$; **$p < 0.01$; ***$p < 0.001$, as determined by one-way ANOVA with Dunnett's multiple comparison test). The mouse icon used for drawing the schematic diagram was retrieved from Flaticon (www.flaticon.com). Scale bars: 100 μm (H&E staining); 20 μm (in situ zymography, TUNEL, and DHE staining).

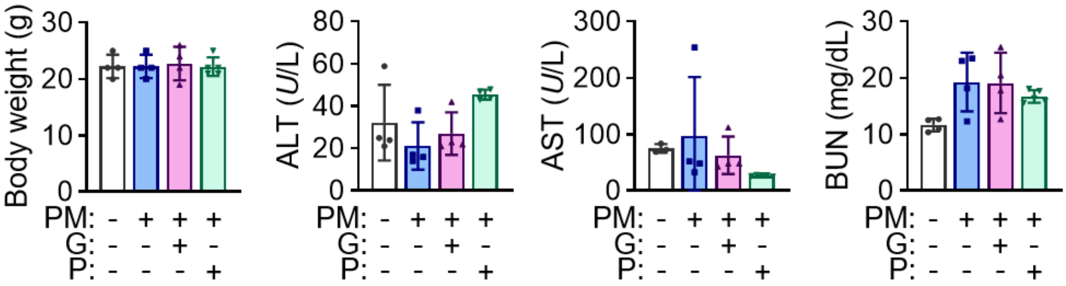

repository**Fig. 23. Minimal in vivo toxicity of gaylussacin and pinosylvic acid.** FVB mice were exposed to PM twice weekly for four weeks by intratracheal instillation with or without gaylussacin (G; 40 mg/kg) or pinosylvic acid (P; 24.48 mg/kg, equimolar to 96 μmol/kg) administered orally once daily, five days/week. Body weight and serum ALT, AST, and BUN levels were measured (mean ± SD, *n* = 3–5). No statistically significant differences were observed (as determined by one-way ANOVA with Tukey's multiple comparison test).
